# Effectiveness of Educational and Psychoeducational Self-Management Interventions in Children and Adolescents With Type 1 Diabetes: A Systematic Review and Meta-Analysis

**DOI:** 10.1155/2024/2921845

**Published:** 2024-10-08

**Authors:** Emma. J. Cockcroft, Ross Clarke, Renuka. P. Dias, Jenny Lloyd, Robert H. Mann, Parth Narendran, Charlotte Reburn, Ben Smith, Jane R. Smith, Robert. C. Andrews

**Affiliations:** ^1^University of Exeter Medical School, EX1 2JP, Exeter, UK; ^2^Department of Paediatric Endocrinology and Diabetes, Birmingham Women and Children's NHS Foundation Trust, B4 6NH, Birmingham, UK; ^3^Institute of Metabolism and Systems Research, University of Birmingham, B15 2TT, Birmingham, UK; ^4^Department of Diabetes, University Hospitals Birmingham NHS Foundation Trust, B4 6NH, Birmingham, UK; ^5^NIHR Exeter Biomedical Research Centre (BRC), EX1 2JP, Exeter, UK

## Abstract

**Aim:** Type 1 diabetes (T1D) is one of the most common chronic conditions in children and adolescents. Approximately 1.5 million young people are currently living with T1D throughout the world. Despite recent improvement in overall indices of metabolic control in children and adolescents with T1D, control remains suboptimal and additional approaches are needed. The aim of the study was to conduct a systematic review and meta-analysis of educational and psychoeducational self-management interventions, to help optimize future interventions including physical activity support.

**Methods:** A systematic review and meta-analysis were conducted according to our registered protocol (PROSPERO CRD42022295932) and are reported in line with the PRISMA 2020 guidance. We searched five databases (MEDLINE, EMBASE, PsycINFO [via Ovid], CINAHL [via EBSCO], Cochrane Library) from 1994 up to May 2024. We included randomized controlled trials assessing the effectiveness of self-management interventions. Outcomes of interest included HbA1c and quality of life (QoL) as well as self-care behaviors, diabetes knowledge, and self-efficacy. Meta-analyses were conducted using a random effects model.

**Results:** In total, 46 papers were included, reporting on 30 interventions. Meta-analyses showed small short-term improvements in HbA1c (MD = −2.58 mmol/L, 95% CI −4.44 to −0.71, *p*=0.007) and QoL (mean difference [MD] = 1.37, 95% CI 0.19–2.54, *p*=0.02). Prespecified subgroup analyses suggested no significant difference in effectiveness of psychoeducational and education-only interventions. Quality of included studies was low with 27 having a high risk of bias.

**Conclusion:** There is a lack of robust evidence that current self-management interventions result in clinically meaningful improvements in HbA1c and QoL. Future research should focus on redefining approaches to supporting and encouraging self-management.

## 1. Introduction

Globally, Type 1 diabetes (T1D) is one of the most common chronic conditions in children and adolescents [1]. Approximately 1.5 million people under the age of 20 are living with T1D in 2021 throughout the world [2]. The prevalence of T1D is rising, with the global in the incidence rate increasing by approximately 3% per annum [3]. There is currently no cure for established T1D. Instead, people living with T1D use exogenous insulin, combined with monitoring and management of blood glucose, dietary intake, and physical activity.

Provision of self-management education (SME), sometimes coupled with psychological support (i.e., psychoeducation), is a key element in the care of people living with T1D, alongside regular clinical support. SME refers to a category of educational interventions that help individuals living with a chronic disease to manage their condition to achieve the best possible quality of life (QoL). SME is interactive and focuses on building skills such as goal setting, decision making, problem solving, and/or self-monitoring [4]. T1D requires self-management by the patient, including calculating insulin doses, carbohydrate counting, physical activity, and hypoglycaemia management. The 2022 International Society for Pediatric and Adolescent Diabetes (ISPAD) guidance [5] states that education around these principles should be provided to all children and adolescents living with T1D and their families. Information should be comprehensive, age-appropriate, and tailored to meet the patient's and their family's needs. Guidance suggests each multidisciplinary team needs to construct their own approach, with no universal or national programs available to children and adolescents. This approach may add burden to the workload of healthcare teams, as well as increasing potential for nonevidence-based and suboptimal approaches being implemented within clinics.

There have been several systematic reviews assessing the effectiveness of self-management interventions in children and adolescents living with T1D, recently summarized in an umbrella reviewed by Rohilla et al. [6]. These reviews vary in quality and scope. They include a wide age range (up to age 30 years) and focus on specific intervention formats, such as digital interventions (14). Rohilla et al. [6] concluded that there was inconsistent evidence on the effectiveness of self-management interventions, with only a small number of interventions tested among children and adolescents. The most recent review focused on children and adolescents included interventions conducted prior to March 2016 and only included UK studies [7]. This review concluded that there was insufficient evidence to recommend the use of any psychoeducational program for children and adolescents living with T1D. Since 2016, seven trials of self-management education interventions have been published, so there is a need to update these reviews.

Physical activity is a key component in the management of T1D. Despite the potential benefits, many children and adolescents living with T1D are not meeting the recommended levels of physical activity, which is at least 60 min of moderate to vigorous physical activity every day [8]. One of the key barriers to physical activity is a lack of knowledge. Being active requires self-management, in terms of adjustments to insulin or changes to carbohydrate intake. Therefore, any attempts to support physical activity in children and adolescents living with T1D should be included in SME. To date, interventions have focused on a prescriptive (rather than a supportive) approach to enabling physical activity (12), with limited emphasis on knowledge exchange and/or self-management support. There is a need for either self-management interventions to include a greater focus on supporting physical activity or more holistic approaches to physical activity interventions, integrating other elements of SME.

The overarching aim of this review is to systematically review and synthesize existing literature to determine the effects of educational and psychoeducational self-management interventions on the health and well-being of children and adolescents (<18 years) living with T1D. In turn, this will help to inform the integration of evidence-based SME into pediatric clinics, as well as guiding optimization of future interventions to include physical activity support.

## 2. Methods

The protocol for this review was registered via the International Prospective Register for Systematic Reviews (PROSPERO Registration Number: CRD42022295932) and is reported in accordance with the Preferred Reporting Items of Systematic Reviews and Meta-Analysis (PRISMA) guidelines (Supporting information S1: File 1) [9].

### 2.1. Search Strategy

Five databases—MEDLINE, EMBASE, PsycINFO (via Ovid), CINAHL (Via EBSCO), and Cochrane Library—were systematically searched up to May 2024 for relevant citations published from 1994, corresponding with developments in research on T1D over the last three decades which have led to large increase in the flexibility of treatments, specifically those resulting from the diabetes control and complications trial [10]. Search strategies were developed by the lead author (EC), with assistance from an information specialist, members of the research team, and the Young Persons Advisory Group (YPAG) for the wider program of work to which this review links. Free-text and medical subject heading (MeSH) terms were combined using Boolean operators “OR” and “AND” to develop a comprehensive search relating to the population and intervention of interest (see Supporting information S2: File 2 for detailed search terms). Searches were developed for MEDLINE and adapted as appropriate for other databases (https://sr-accelerator.com/#/polyglot).

### 2.2. Eligibility Criteria

We included randomized controlled trials (RCTs) that examined the effectiveness of SME interventions in children and adolescents living with T1D. We defined children and adolescents as from birth up to and including those aged 18 years. We included any interventions targeting children and adolescents (rather than parents/carers or health professionals alone) that aimed to improve children's and/or adolescents' skills in or understanding of one or more of the key T1D self-management behaviors including diet, insulin dosing, glucose monitoring, and physical activity. Education could be delivered alone (i.e., educational interventions) or in combination with psychological components designed to support coping with emotional aspects of diabetes (i.e., psychoeducational interventions). Purely psychological interventions (e.g., cognitive behavioral therapy alone) were excluded due to our primary focus on education regarding self-management. Interventions could be delivered in any modality, setting, and location, provided they were intended to support patients with self-management.

Studies had to be RCTs or cluster RCTs that involved a nonintervention, attention control, or “usual care” arm. Studies combining T1D and Type 2 diabetes, or including adults (> 18 years), were excluded.

### 2.3. Types of Outcome Measures

The primary outcomes of interest were glycaemic control (as measured by HbA1c) and QoL. Secondary outcomes included self-efficacy, self-management behaviors, and diabetes knowledge.

### 2.4. Study Selection and Data Extraction

Retrieved citations were uploaded into the review management system Covidence (Veritas Health Innovation), and duplicates were removed. At least two independent reviewers (EC, BS, CR, and RM) completed screening for both titles/abstracts and full-text articles. Disagreements were resolved by consensus or discussion with a third reviewer, as necessary.

Data extraction was undertaken by one reviewer (EC or BS) and checked by a second reviewer for accuracy using a prepiloted data collection form in Covidence. Data were extracted on study design (RCT or cluster RCT), participant (i.e., age), intervention characteristics (i.e., format), behavior change techniques (BCTS) utilized, and description of control condition. We also extracted data on sample size, participant characteristics, and baseline and follow-up outcome data for each trial arm. Authors of included studies were contacted by email for clarification on trial methods or data whenever there was insufficient information reported. A total of three authors were contacted [11–13], with two providing further clarification [12, 13].

Quality assessment was completed by one reviewer (EC or BS) and checked by a second reviewer with disparities during the checking process resolved through discussion. Quality of individual trials was assessed using six domains of the Cochrane Collaboration's tool for assessing risk of bias [14], including sequence generation; allocation concealment; blinding of outcome assessors; completeness of outcome data; selective reporting of outcomes; and other sources of bias. For each domain, studies were classified as being at low, high, or unclear risk of bias.

A single reviewer (EC or BS) used the BCTTv1 taxonomy [15] to identify and code BCTs used in interventions into the 16 overarching domains. This was checked by a second reviewer (EC or BS).

### 2.5. Data Synthesis and Analysis

Meta-analyses based on mean differences (MD) for primary outcomes were conducted in Review Manager (RevMan; version 5.4, The Cochrane Collaboration, 2020) using random effects model. We used Cochran's *I*^2^ statistic to assess heterogeneity with 0%, 25%, 50%, or 75% indicating no, low, moderate, or high heterogeneity, respectively. We conducted sensitivity analysis to explore heterogeneity by excluding one study at a time. Any potential publication bias was assessed by visual inspection of the funnel plot for the HbA1c outcome.

In alignment with the Cochrane Handbook, where only medians and interquartile ranges were reported, these were converted following the methods of Wan et al. [16].

We analyzed outcomes reported at the end of intervention (up to and including 3-month follow-up) and longer term at 6 or more months postintervention.

Subgroup analysis was performed to assess effectiveness based on intervention type (education vs. psychoeducation).

### 2.6. Patient and Public Involvement

This systematic review was conducted in collaboration with a YPAG for the wider program of work to which this review links. The group includes four young people living with T1D and four parents/carers of young people living with T1D. The YPAG contributed to the development of the project aims, search strategy, and in the interpretation of data.

## 3. Results

**Figure 1:**
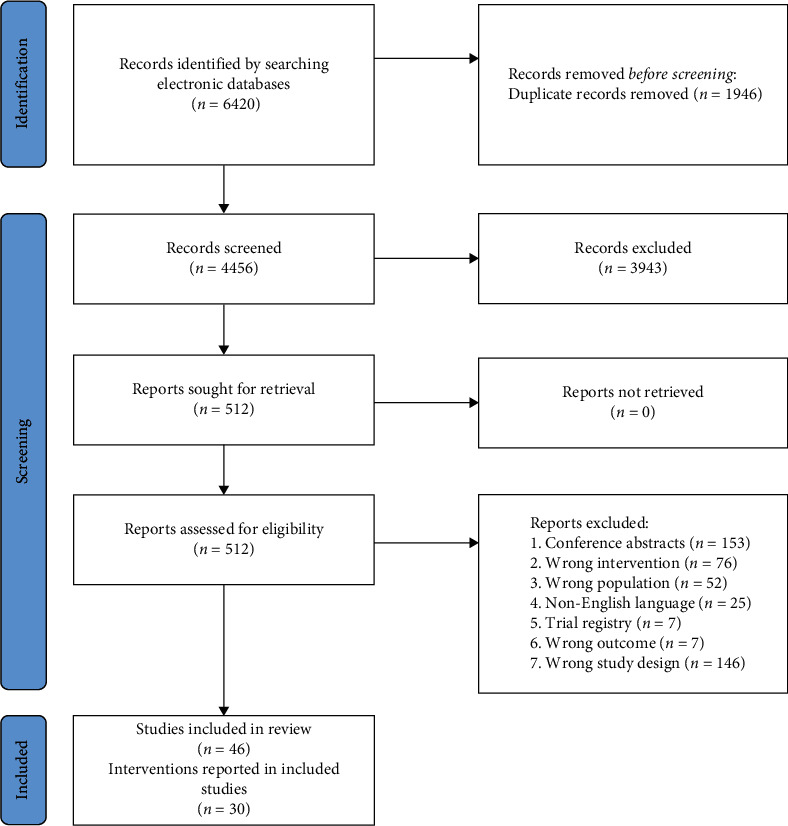
Preferred reporting for systematic reviews and meta-analyses (PRISMA) flowchart of the literature search and article selection.

Of 4456 unique records screened, 30 interventions met the inclusion criteria, comprising 28 RCTs and two cluster RCTs (see Figure 1). Five interventions were conducted in the United Kingdom, seven in other parts of Europe, 15 in North America, two in Asia, and one in Africa. Findings from included studies are summarize in Table 1.

### 3.1. Risk of Bias

**Figure 2:**
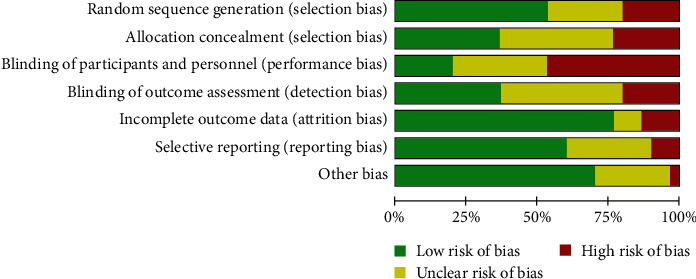
Results of the risk of bias analysis.

Figure 2 outlines the risk of bias in the included studies. The quality of the included studies was low, with 27 studies deemed to be at high risk of bias and three studies having low risk of bias [12, 31, 45]. The main areas where high risk of bias was observed was for blinding, of both participants (sometimes unavoidable in behavioral interventions) and data assessors.

### 3.2. Participant and Intervention Characteristics

Characteristics of the participants and interventions are described in Tables 2 and 3. Fourteen interventions were classified as educational, and 16 as psychoeducational. The number of participants in the included studies ranged from 16 [19] to 475 [27]. Twelve of the 30 studies had > 200 participants. Most studies (*n* = 17) included both children (> 11 years) and adolescents (11–18 years) with the remaining 13 studies including only adolescents. No studies focussed solely on children under 11 years.

Most interventions were delivered in person (*n* = 22). Others were delivered via websites (*n* = 3), video games (*n* = 2), text messaging (*n* = 1), videos (*n* = 1), and apps (*n* = 1). Interventions were mainly delivered to the young person one-on-one (*n* = 12), or to a family unit (*n* = 8). Other delivery formats included groups of young people (*n* = 6) or groups of families (*n* = 4). Interventions varied in length from a one-off session [24] to an intervention with multiple sessions delivered over 24 months [42].

Of the included interventions, nine explicitly mentioned the involvement of parents and/or young people in development of the intervention and 13 reported that interventions were informed by theory. Most BCTs employed were categorized under four behavior change domains, namely shaping of knowledge (*n* = 30), feedback and monitoring (*n* = 15), goals and planning (*n* = 12), and social support (*n* = 12) (Table 4).

The in person interventions (*n* = 22) were commonly delivered by a nonspecified member of the clinical care team (*n* = 5), or a nurse (*n* = 3), dietician (*n* = 1), or psychologist (*n* = 1). Interventions which were not delivered by clinical staff were delivered by generic providers (*n* = 6), often a graduate researcher or other member of research team, a robot (*n* = 1), or did not report this (*n* = 5).

Control conditions were most often usual care (*n* = 18), with others using attention control groups (*n* = 8) or no intervention (*n* = 4). Details on control conditions are included in Table 1.

### 3.3. Glycaemic Control

Of the 30 studies, 20 assessed the effect of interventions on HbA1c. Nineteen of these studies provided appropriate data, on a total of 2812 participants, suitable for inclusion in a meta-analysis [13, 20–23, 26, 27, 29–31, 33, 38, 39, 41–44, 46, 47]. One study that was not included in the meta-analysis, as it did not report standard deviations, found no differences in HbA1c across the study groups [32]. Two studies were assessed as having low risk of bias [13, 31].

**Figure 3:**
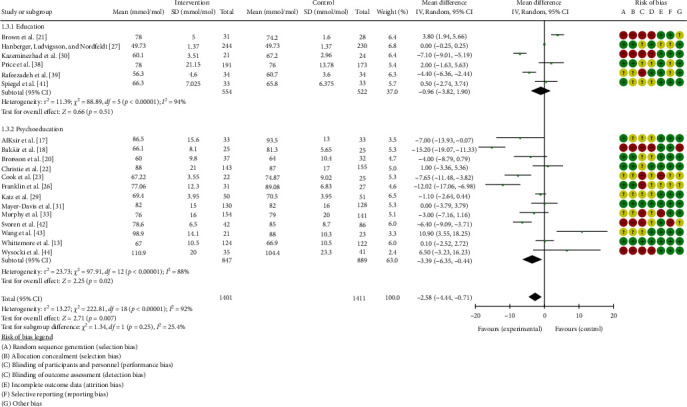
Meta-analysis of the effects of educational and psychoeducational interventions on HbA1c.

Compared with control conditions, educational and psychoeducational interventions reduced HbA1c (mean difference (MD) = −2.58 mmol/L, 95% confidence interval (CI) = −4.44, −0.71, *p*=0.007), see Figure 3. Overall, interventions classified as psychoeducational reduced HBA1c to a greater extent (14 studies, MD = −3.39 mmol/L, 95% CI −6.35, −0.44) than educational interventions (six studies, MD = −0.96 mmol/L, 95% CI −3.82, 1.90), though subgroup analysis suggested no significant between group differences (*p*=0.25). The studies were highly heterogeneous (*p*  < 0.001, *I*^2^ = 92%). Sensitivity analysis indicated no significant alteration of results from excluding individual studies, suggesting heterogeneity was not a result of any individual studies.

**Figure 4:**
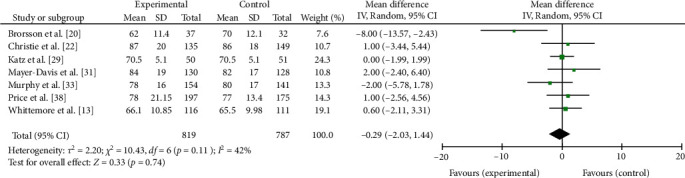
Meta-analysis of the effects of educational and psychoeducational intervention on HbA1c at 6 or more months postintervention follow-up.

There was no pooled effect across seven studies assessing HbA1c at longer term follow-up 6 or more months postintervention (MD = −0.29 mmol/L, 95% CI −2.03, 1.44, *p*=0.74), see Figure 4. Long-term follow-up ranged from 6 [13] to 24 months [22].

Assessment of publication bias showed symmetry of the funnel plot, suggesting no evidence of publication bias across studies included in the meta-analysis for HbA1c.

### 3.4. General QoL

**Figure 5:**
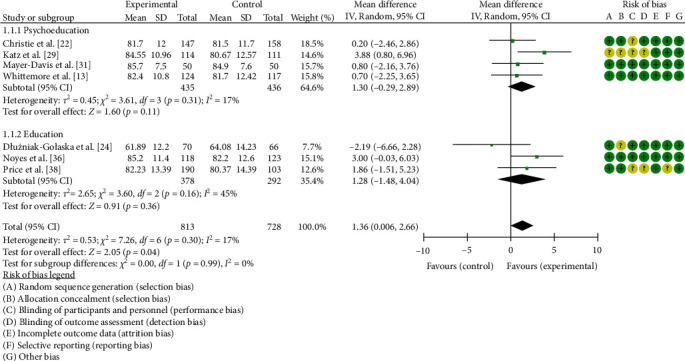
Meta-analysis of the effects of educational and psychoeducational intervention on QoL.

Of 30 studies, seven (involving 1541 participants) assessed the effect of interventions on QoL using the PedsQL scale [13, 22, 24, 29, 31, 36, 38] and were included in the meta-analysis. Studies were generally of good quality with three having low risk of bias. Compared with control, educational, and psychoeducational interventions improved QoL (MD = 1.37, 95% CI 0.19, 2.54, *p*=0.02), see Figure 5. Subgroup analysis suggested no significant difference in effects on QoL (*p*=0.84) between educational (MD = 1.54, 95% CI −0.47, 3.55) and psychoeducational interventions (MD = 1.28, 95% CI −0.17, 2.73).

**Figure 6:**
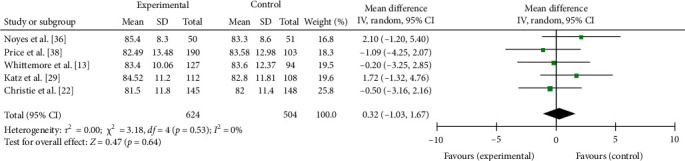
Meta-analysis of the effects of educational and psychoeducational intervention on QoL at 6 or more months postintervention follow-up.

Across five studies including a longer term follow-up 6 or more months postintervention, there was no pooled effect on QoL (MD = 0.32, 95% CI −1.03, 1.67, *p*=0.64), see Figure 6. Long-term follow-up ranged from 6 [12, 13] to 24 months [22, 29].

Two additional studies of educational interventions measured QoL using alternative scales (DISABKIDS [20]; World Health Organisation QoL questionnaire [WHOQOL-BREF] [48]) but neither found differences between groups.

### 3.5. Diabetes-Related QoL

Four studies measured diabetes-related QoL [12, 22, 33, 38], three of which used the PedsQL diabetes module, and one of which used the Diabetes Quality of Life Youth Scale (DQOLY-SF) [33] for assessment. Three studies found no effect of interventions on diabetes-related QoL [22, 33, 38] and one study found a decrease, indicating a negative effect of the intervention [12]. Three of these studies had low risk of bias [12, 22, 38], and one had high risk [33].

### 3.6. Secondary Outcomes

#### 3.6.1. Diabetes Self-Management Tasks

##### 3.6.1.1. Physical Activity

Two studies, both with high risk of bias and relatively small sample sizes, measured physical activity. One study [40], using the Godin–Shephard Leisure-Time Physical Activity Questionnaire [49], found an effect of the intervention on physical activity levels (*p* > 0.05). The other study [39] found that adherence to an exercise regimen (measured using a 20-item researcher-made Diet and Exercise Regimen Adherence Questionnaire) was significantly higher in the intervention group (*p*=0.04).

##### 3.6.1.2. Diet

Two studies, both with high risk of bias, measured self-management relating to diet. One [34] found a positive effect (*p*=0.015) at 18 months, with a 7.2 point improvement to Healthy Eating Index−2005 (HEI2005) (mean ± SE 64.6 ± 2.0 versus 57.4 ± 1.6) in the intervention versus control group. Using a 20-item researcher-made Diet and Exercise Regimen Adherence Questionnaire, the other study [39] found that in a group of 68 children, scores for adherence to diet regimen were significantly higher in the intervention compared to the control group (intervention 38.6 ± 45.7, control 35.1 ± 5.7, *p*=0.01).

##### 3.6.1.3. Insulin Administration

Three studies, all with high risk of bias, assessed insulin administration as an outcome using various measures—diabetes social support interview (DSSI) [26]; scores for adherence to the medication regimen [39]; and insulin dose adjustment behavior [33]. None showed positive effects of the interventions on insulin administration.

##### 3.6.1.4. Glucose Monitoring

Three studies [27, 29, 32], all with high risk of bias, measured glucose monitoring (e.g., times/day) and found no difference between groups. One study [23] found that intervention group participants were doing blood glucose testing more often than controls and another [26], using DSSI, reported a positive effect of the intervention on blood glucose monitoring.

##### 3.6.1.5. Self-Management Scales

Six studies assessed self-management behavior using general scales [11, 13, 23, 31, 44], including the Diabetes Self-management Assessment Profile; Self-Care Inventory Scale; Diabetes Problem-Solving Measure for Adolescents; Self-Management of T1D in Adolescence; the Diabetes Behavior Rating Scale; and the Transition Readiness Assessment Tool. Four of these six studies found no effect of the interventions on self-management [11, 13, 23, 44], but in the other two [31, 46], the interventions were associated with improvements in self-management. Only one study assessing general self-management had low risk of bias [31]

#### 3.6.2. Diabetes knowledge

Six included studies reported outcomes related to diabetes knowledge [19, 21, 28, 37, 41, 47]. All studies had high risk of bias. Two out of four of these studies assessing general diabetes knowledge through tests, interview questions, and questionnaires found increased knowledge in the intervention compared to control groups [28, 47] and two studies found no improvement [19, 21].

Two additional studies, using the PedsCarb quiz [37] and carbohydrate counting accuracy questionnaire [41], found no effect of the interventions on knowledge of carbohydrate counting.

#### 3.6.3. Self-Efficacy

Six of the included studies assessed self-efficacy [11, 13, 20, 21, 26, 47] using questionnaires, including the diabetes management self-efficacy scale [47]; Swe-DES 23 [20]; and self-efficacy for diabetes scales [11, 13, 21, 26]. Only two of these [26, 47] reported improvements as a result of the intervention. All studies had high risk of bias.

## 4. Discussion

To our best knowledge, this is the first review to systematically assess evidence from across the world on the effectiveness of both education and psychoeducational interventions specifically for children and adolescents with T1D. Previous reviews have limited interventions to a UK context [7], have focused on particular types of interventions such as technology-based [50] or telemedicine [51], or included interventions for both adults and children/adolescents [52]. Most similar are the systematic reviews by Murphy et al. [53] and Hampson et al. [54], conducted in 2006 and 2001, respectively. Considering ongoing research over the last two decades, the current review updates and expands this existing evidence base.

Results stemming from the 30 included RCTs suggest limited effects of these interventions on clinical and psychological outcomes. Included studies were mostly of poor quality, precluding firm conclusions. Pooled data from 20 studies, and particularly 14 studies of psychoeducational interventions, showed small, statistically significant short-term impacts on glycaemic control, but with minimal sustained clinical importance. Thresholds for clinical benefit vary depending on source but are estimated at around 11 mmol/mol [55], with the National Institute for Health and Care Excellence acknowledging a change of 5.5 mmol/mol as clinically significant [56]. The small effect in the current review is in line with previous reviews [51, 52]. For example, findings from children and adolescents in a review on telemedicine suggested an overall reduction of 0.84 mmol/mol [51], and another review of behavior programs showed a reduction of 1.88 mmol/mol at 6 months postintervention [52].

Pooled data from seven studies suggested statistically significant improvements in general QoL from SME, but likely below the threshold to be considered clinically meaningful. The pooled improvement of +1.37 in general QoL is also unlikely to be clinically meaningful, with research suggesting that a threshold of 4.72 represents a meaningful difference in the PedsQoL measure used [57]. There is also no evidence for sustained improvement in trials with longer term follow-up. Previous reviews including studies targeting both adults and children have similarly shown limited effects of telemedicine [51] or behavioral programs [52] on QoL. Specifically in children and adolescents, Kazemi et al. [58] found mixed effects of peer-based interventions on health-related QoL. It is worth noting that only nine of the 30 studies included in our review reported QoL assessment, with seven using the generic PedsQoL tool, and four using diabetes-related QoL measures. Although measures such as HbA1c are used clinically, QoL provides better insight into how a new intervention may affect a patient's life. Therefore, future studies should ensure that a standardized and validated measure of QoL is included to help inform decisions about the suitability of an intervention. There is a current absence of validation studies and consensus on the most appropriate measures [59].

There was more limited evidence and studies showed mixed results in terms of other outcomes, including diabetes-related QoL, self-management behaviors, diabetes knowledge, and self-efficacy. For outcomes where there was the most evidence (maximum six studies), most studies suggested limited effects.

Although there were no statistically significant differences from subgroup analyses examining the effects of the different types of interventions on HbA1c, there is some indication that a combined approach offering education with psychological support may be favorable and should be considered in future intervention development. Traditional education programs aim to teach diabetes knowledge and skills, and this can be supplemented with psychological components and techniques to provide support for behavior change, for example, by enhancing motivation and encouraging goal setting, problem-solving, and coping with emotional impacts and setbacks. The importance of psychological elements in addition to education has been acknowledged in recent ISPAD guidance [60], which now emphasize incorporating goal setting, problem-solving, motivational interviewing, communication skills training, family conflict resolution, development of coping skills, and stress management into more traditional education only programs. Related to this, the concept of self-efficacy (i.e., the confidence that one can carry out a behavior and anticipated consequences of that behavior required to reach a desired goal) is likely to be important in diabetes SME as a mediator between knowledge and performance of self-care behaviors [61]. Integrating approaches to boost self-efficacy and the four sources of self-efficacy (i.e., mastery experiences; vicarious experiences; verbal persuasion; and physiological and affective states [62]) offers the potential to improve the effectiveness of interventions and health outcomes [63, 64]. Of the interventions included in this review, six included self-efficacy as an outcome, but only two of these showed improvements [26, 47]. These findings showing limited effects on self-efficacy reflect those from reviews by Knox et al. [65], who showed mixed results for technology-based interventions, and Charalampopoulos et al. [7], who showed no effect of United Kingdom based psychoeducational interventions on self-efficacy. The importance of self-efficacy was emphasized by our project's YPAG and should be considered when designing future self-management intervention studies as both a key mediator to target in changing self-management behaviors and as an outcome to assess.

It is worth highlighting that over half of the included interventions were delivered to participants spanning chronological ages representative of both children and adolescents (e.g., 8–18 years) and different developmental stages. These ages and stages would require different learning styles and intervention needs. The reported interventions do not differentiate the effects of different developmental stages (or ages) meaning we are unable to assess if these interventions may have been effective in older participants. Future interventions should consider more targeted approaches based on educational psychology as well as the needs and preferences of different age groups.

Our review highlights that most current interventions focus primarily on BCTs related to shaping knowledge. Given their limited effects, it is important to develop interventions which include other categories of BCTs, including those shown to relate to improving self-efficacy, such as goal setting and self-monitoring, as well as drawing on theory relating to developing self-efficacy. This is an important consideration for future interventions as despite guidance explicitly stating that patient and public involvement and theory were essential elements in developing interventions, only four of the included studies used both [66].

This study has several limitations which should be considered. First, because of varying intervention designs, significant heterogeneity existed between included studies and warrants consideration when interpreting the findings and future exploration. Meaningful moderator and subgroup analyses could not be performed to explore whether differences in, for example, intervention duration and intervention delivery mode accounted for the observed heterogeneity, because the number of studies was small. Caution should also be used when interpreting findings for secondary outcome measures as studies were unlikely to be powered appropriately to detect these and the limited number of studies for each outcome precluded meta-analyses. Second, only published articles in the English language were included. Therefore, relevant studies in other languages may have been missed. Additionally, our analysis of BCTs was limited by brief intervention descriptions, resulting in using overarching BCT categories rather than coding-specific techniques, limiting further analysis, and detailed discussion of effective BCTs. Lastly, the timeframe of the included studies requires consideration. More specifically, there have been significant changes in management of T1D throughout the past 30 years (i.e., timeframe for this review), including insulin pump therapy, continuous glucose monitoring, and emerging closed-loop therapy. Therefore, review findings should be considered in the context of this changing landscape.

## 5. Conclusion

Our review suggests that current evidence does not support the use of existing programs as a means of bringing about clinically meaningful improvements in self-management or health outcomes in children and adolescents living with T1D. In light of this, we suggest that future interventions should be codeveloped with key stakeholders, including young people, parents/carers, and clinicians, and be theory-informed to more effectively support the behavioral changes required for effective self-management of T1D. Key mediators of self-care behaviors, such as self-efficacy, should be effectively targeted using appropriate BCTs to supplement more formal education providing knowledge and skills, and delivered in a format acceptable to stakeholders. Trials should use validated measures of QoL, both generic and diabetes-specific, such as PEDSQL and PEDSQL-diabetes, to consider impacts on patients and their families beyond clinical markers.

## Figures and Tables

**Table 1:** Summary of findings of included studies.

First author, year	Number of participants (baseline)	HbA1c	QoL	Self-efficacy	knowledge	Self-management	Key findings
Alksir et al., 2022 [17]	66	**↓**	—	—	—	**↑**	• Significant increase in TRAQ • Significant reduction in HbA1c

Bakir et al., 2020 [18]	50	**↓**	—	**↑**	**↑**	—	• Improvements in knowledge levels, personal motivation, social motivation levels, and behavioral skills • HbA1c levels decreased significantly

Bernier et al., 2018 [19]	16	—	—	—	↔	—	• Knowledge gains no different between groups (DKT2)

Brorsson et al., 2019 [20]	69	**↓**	**↔**	**↔**	—	—	• Improvements in glycaemic control at 12 months • No effects on self-perceived health, health related quality of life (QoL), family conflicts, and self-efficacy

Brown et al., 1997 [21]	59	**↔**	—	↔	↔	—	• No difference in groups in knowledge about diabetes or HbA1c • No significant difference in self-efficacy

Christie et al., 2016 [22]	288	**↔**	↔	—	—	—	• Intervention did not improve HbA1c at 12 months or 24 months • No significant changes in QoL (generic and diabetes module)

Cook et al., 2002 [23]	53	**↔**	—	—	—	**↑**	• No between group difference in problem solving, HbA1c • Significantly more blood glucose tests/day in experimental group at 6 months

Dłużniak-Gołaska et al., 2020 [24]	170	—	↔	—	—	—	• No significant changes in QoL at 6 months post intervention

Fiallo-Scharer et al., 2019 [25]	214	**↔**	↔	—	—	—	• No effect of intervention on QoL or HbA1c

Franklin et al., 2006 [26]	92	**↔**	—	**↑**	—	**↑**	• Improvements in diabetes self-efficacy and self-reported adherence • No change in HbA1c

Hanberger et al., 2013 [27]	474	**↔**	↔	—	—	**↔**	• No difference between groups in terms of QoL • No effects HbA1c, frequency of blood glucose self-control, severe hypoglycaemia

Henkemans et al., 2017 [28]	28	—	—	—	**↑**	—	• Increased in diabetes knowledge

Holmes et al., 2014 [11]	266	**↔**	↔	↔	—	↔	• No significant difference in HbA1c • No different in psychosocial outcomes (self-efficacy, QoL, diabetes behavior rating)

Katz et al., 2014 [29]	153	**↔**	↔	—	—	—	• No difference in HbA1c or QoL

Kazeminezhad et al., 2018 [30]	50	**↔**	—	—	—	—	• No difference in FBS or HbA1c between control and experimental groups

Mayer-Davis et al., 2018 [31]	258	**↔**	**↑**	—	—	**↑**	• HbA1c not significantly different between intervention and control • Intervention was associated with improved motivation, problem solving, diabetes self-management profile, and QoL

McGill et al., 2020 [32]	301	**↔**	—	—	—	↔	• No significant difference in HbA1c or BG monitoring

Murphy et al., 2012 [33]	305	**↔**	↔	—	—	↔	• HbA1c, QoL, well-being, family responsibility, and insulin dose adjustment behaviors were comparable between groups

Nansel et al., 2015 [34]	136	**↔**	—	—	—	**↑**	• Positive effect of intervention on dietary outcomes (HEI2005 and WPFD) • No effect on HbA1c

Nordfeldt et al., 2003 [35]	332	**↔**	—	—	—	—	• Decrease in yearly incidence of severe hypoglycaemia • No change in HbA1c

Noyes et al., 2014 [36]	293	**↔**	↓	—	—	—	• No evidence of change in HbA1c • Treatment adherence favoured control condition (at 6 months) • Increased worry in the EPIC kit group

Pais et al., 2021 [37]	50	—	—	—	↔	—	• Change in carbohydrate counting accuracy a 3 months postintervention were not significant

Price et al., 2016 [38]	370	**↔**	**↑**	—	—	—	• The intervention group significant improved QoL (general) but not diabetes specific QoL • No significant difference in HbA1c

Rafeezadeh et al., 2019 [39]	70	**↔**	—	—	—	**↔↑**	• No significant difference between intervention and control for HbA1c or adherence to medication regimen • Scores for adherence to diet and exercise regimen significantly higher in the intervention group compared to control

Skoufa et al., 2023 [40]	84	—	↔	—	—	↔	• No significant difference in physical activity levels or QoL

Spiegel et al., 2012 [41]	66	**↔**	—	—	**↔**	—	• No difference in HbA1c or carbohydrate counting accuracy

Svoren et al., 2003 [42]	301	**↔**	—	—	—	—	• Significantly reduced rates of short-term adverse outcomes • No significant difference in follow-up HbA1c

Whittemore, 2012 [13], United States	320	**↔**	↔	↔	—	↔	• No effects on QoL, Self-management, self-efficacy • No effects on HbA1c

Wang et al., 2010 [43]	54	**↔**	↔	—	—	↔	• HbA1c At 6 months lower in the control (Structured education) group • CES-D Depression Scale, EDIC-QoL, and the summary of diabetes self-care—no difference between groups

Wysocki et al., 2000 [44]	119	**↔**	—	—	—	**↔**	• BFST yielded more improvement in parent-adolescent relations and reduced diabetes-specific conflict • No effects on treatment adherence, HbA1c or self-care

*Note:* **↓** indicates reduction, **↑** indicates increase and **↔** no change with intervention compared to control group.

Abbreviations: BFST, Behavioral-Family Systems Therapy; BG, blood glucose; CES-D, Centre for Epidimioloy Studies Depression Scale; DKT2, Diabetes Knowledge Test 2; EDIC-QOL, Epidemiology of Diabetes Interventions and Complications Quality of Like Questionaire; EPIC, Evidence into Practice—Information Counts; HbA1c, glycated haemoglobin; HEI2205, Healthy Index 2005—measuring conformance with 2005 US guidance; IBM, information-motivation behavioural skills; QoL, Quality of Life; TRAQ, Transition Readiness Assessment Questionnaire; WPFD, whole plant food density.

**Table 2:** Summary of characteristics of 30 studies included in the systematic review.

First author, year	Country (study name)	Intervention type	Control condition	Mode of delivery	Communication method(s)	Intervention description	Intervention duration (months)	Time spent on each session (number of sessions)	PPI	Theory	Number of participants (baseline)	Age range (years) or mean (SD)
Alksir et al., 2022 [17]	Tunisia	Psychoeducation	Usual care	Individual	In person	In person Motivational interviewing-based interventions focused on self-management skills. web-based videos and brochures	6 months	30 (2) + 20 (4)	Y	N	66	13–18

Bakir et al., 2020 [18]	Turkey	Psychoeducation	No intervention	Individual	In person	Nurse home visits to apply components of IBM model	6 months	90 (4) + 15 (5)	N	Y	50	14.6 ± 2.14

Bernier et al., 2018 [19]	USA (NODE)	Education	Standard DSMES control group—nurse education	Individual family dyads	Web-based	Animation-based educational web application for type 1 diabetes mellitus patients—designed to complement standard DSMES	Unclear—during hospitalization	8 online modules	Y	N	16	4–15

Brorsson et al., 2019 [20]	Sweden (GSD-Y)	Education	Group-based standard insulin pump introduction program	Groups of families	In person	Person centered communication and reflection education	5 months	120 (7)	N	Y	69	12–18

Brown et al., 1997 [21]	USA (Packy and Marlon)	Education	Entertainment video game with no health content	Individual	Interactive video game	Video game designed to improve a young person's self-confidence, ability, and motivation to undertake the rigorous self-care necessary to control insulin-dependent diabetes	Unclear—measures at 3 and 6 months after receiving game	Varied “as much or as little as they wished”	N	Y	59	8–16

Christie et al., 2016 [22]	UK (CASCADE)	Psychoeducation	Standard care	Groups (parents and children)	In person	Clinic-based structured educational group incorporating psychological approaches	4 months	120 (4)	Y	N	288	8–16

Cook et al., 2002 [23]	USA (Choices)	Psychoeducation	Routine care	Groups of children and adolescents	In person	Small group problem-solving diabetes self-management education program for adolescents with T1D	1.5 months	120 (6)	N	N	53	13–17

Dłużniak-Gołaska et al., 2020 [24]	Poland	Education	“Traditional” 30 min lecture on nutrition in diabetes	Groups of children and adolescents	In person	“Modern” methods (an interactive quiz/multimedia application) were used alongside traditional education methods (lecture)	n/a. One off session	90 (1)	N	N	170	8–17

Fiallo-Scharer et al., 2019 [25]	USA (T1DSMART)	Education	Usual care	Groups of children and adolescents	In person	Tailored delivery of self-management resources to families' specific self-management barriers. Sessions are delivered in an interactive, small-group format	9 months	75 (4)	Y	N	214	8–16

Franklin et al., 2006 [26]	UK (Sweet talk)	Psychoeducation	Conventional care	Individuals	Text-messaging	Text messages to deliver a theoretically guided behavioral intervention	12 months	100 (3.5)	N	Y	92	8–18

Hanberger et al., 2013 [27]	Sweden, Web 2.0 portal	Education	No access to portal	Individuals	Website	A web portal offering self-directed communication with health professionals, interaction with peers, and access to information	17 months	“Whenever needed and by users' own initiative”	Y	N	474	0–18

Henkemans et al., 2017 [28]	Netherlands	Education	Usual care	Individuals	In person	Personal robot playing diabetes quiz	6 weeks	25 (3)	Y	Y	28	7–14

Holmes et al., 2014 [11]	USA	Psychoeducation; education	Usual care	Individual family	In person	Coping skills training OR diabetes education	12 months	45 (4)	N	N	266	11–14

Katz et al., 2014 [29]	USA (CA + Ultra)	Psychoeducation	Usual pediatric diabetes subspecialty care including basic care coordination by the Care ambassador (CA)	Individual family	In person	CA + ultra participants received a psychoeducational intervention conducted at quarterly study visits, in addition to monthly outreach and quarterly diabetes care and care coordination	12 months	30 (7)	N	N	153	8–16

Kazeminezhad et al., 2018 [30]	Iran	Education	Control—not explicitly specified	Groups of children and adolescents	In person	Group education sessions (theoretical and practical)	3 months	90 (7)	N	N	50	10–18

Mayer-Davis et al., 2018 [31]	USA (FLEX)	Psychoeducation	Attention matched usual care	Individuals	In person	Adaptive behavioral intervention using motivational interviewing and problem solving skills training. Included the FLEX “toolbox” with T1D education, social support, text messaging	18 months	60 (4)	N	Y	258	13–16

McGill et al., 2020 [32]	USA (Teenwork)	Psychoeducation	Routine clinical care	Individuals	In person; phone	Teenwork + text message reminders to check BG	12 months	Duration unclear (5) 1–4 texts/day	N	N	301	13–17

Murphy et al., 2012 [33]	UK (FACTS)	Psychoeducation	Conventional care	Groups of children and adolescents	In person	Group education sessions (4–6 families per group) incorporating conventional diabetes self-management education with family communication training	6 months	90 (6)	Y	Y	305	6–16

Nansel et al., 2015 [34]	USA (WE-CAN)	Psychoeducation	Attention control—equal frequency of contacts with research staff, focused on case management	Individual family	In person	Family-based behavioral intervention with integrated a motivational interviewing style of interaction	15 months	15 (9)	Y	Y	136	8–17

Nordfeldt et al., 2003 [35]	Sweden	Education	Traditional treatment	Individual family	Videos	Self-study material: Two video programs designed to review skills for self-control and treatment, aimed at preventing severe hypoglycaemia	n/a	60 (4)	N	N	332	3–18

Noyes et al., 2014 [36]	UK (EPIC)	Education	Usual treatment	Individuals	In person	Self-management kits to empower children to achieve glycaemic control (kits comprising booklets, magazines, leaflets, CDs, and website links)	6 months	Self-directed	Y	Y	293	6–18

Pais et al., 2021 [37]	Canada (Counting Carbs to Be in Charge)	Education	Standard care (in class education)	Individuals	Website	Internet-based educational module	1 week	Daily text messages	Y	N	50	12–18

Price et al., 2016 [38]	UK (KiCK-OFF)	Education	Usual care (may have included small group education at some centers)	Groups of children and adolescents	In person	Structured education course	5 days	360 (5)	N	Y	370	11–16

Rafeezadeh et al., 2019 [39]	Iran	Education	Not described	Individuals	Video game	Educational interactive video game	3 months	1–4 h per week for 12 weeks	Y	N	70	8–12

Skoufa et al., 2023 [40]	Greece	Education; other: physical activity + education	Control—normal routine	Groups of children and adolescents	In person	Ten-day summer diabetes sports camp including physical activity and educational sessions	10 days	13 h (10)	N	N	84	7–18

Spiegel et al., 2012 [41]	USA (CA+)	Education	Handout and brief (5 min) discussion on carbohydrate counting	Groups of children and adolescents	In person	Carbohydrate counting class with hands on activities and discussion. +Food record feedback sessions	8 weeks	90 (1)	N	N	66	7–16

Svoren et al., 2003 [42]	USA, CA+	Psychoeducation	Standard care	Individual family	In person; phone	Care ambassador assisted family with scheduling apps and monitoring attendance, with addition of delivery of 1–1 psychoeducation modules at clinic appointments	24 months	8(10) + 8(30)	N	N	301	12–18

Whittemore et al., 2012 [13]	USA (TEENCOPE)	Psychoeducation	Attention control—managing diabetes educational program	Individuals	Website	Coping skills internet program	—	—	N	Y	320	11–14

Wang et al., 2010 [43]	USA	Psychoeducation	Structured diabetes education	Individuals	In person	Motivational interviewing-based education	6 months	8 (10)	N	N	54	12–18

Wysocki et al., 2000 [44]	USA (BFST)	Psychoeducation	Standard therapy	Individual family	In person	Ten sessions of behavioral family systems therapy	1.5 Months	10 × BFST sessions	N	N	119	12–17

Abbreviations: DSMES, diabetes self-management education and support; IBM, Information-behavioural-motivation; PPI, Patient and public involvement.

**Table 3:** Participant characteristics of included studies.

First author, year	Sample size	Mean age (SD)	Female (%)	White (%)	Diabetes duration, years (SD)	Using insulin pump (%)
Alksir et al., 2022 [17]	I: 33 C: 33	I: 15.3 (1.65) C: 15.06 (1.71)	NG	NG	I: 6.64 (4.5) C: 4.30 (2.62)	NG
Bakir et al., 2020 [18]	I: 25 C: 25	I: 14.68 (2.14) C: 14.44 (1.56)	I: 52% C: 48%	NG	32% less than 5 years	NG
Bernier 2018 [19]	I: 8 C: 8	10.75 (3.44)	I: 50% C: 75%	I: 87% C: 85%	<48 h	NG
Brorsson et al., 2019 [20]	I: 37 C: 32	I: 14.8 C: 15.1	I: 54% C: 66%	NG	I: 4.5 C: 5.6	NG
Brown et al., 1997 [21]	I: 31 C: 28	Range: 8–16	NG	NG	NG	NG
Christie et al., 2016 [22]	I: 157 C: 158	I: 13.1 (2.1) C: 13.2 (2.1)	I: 57% C: 54%	I: 84% C: 77%	I: 5.7 (3.2) C: 6.1 (3.3)	NG
Cook et al., 2002 [23]	I: 26 C: 27	I: 14.8 (1.17) C: 14.4 (1.36)	I: 50% C: 63%	I: 88% C: 81%	NG	NG
Dłużniak-Gołaska et al., 2020 [24]	I: 70 C: 66	I: 13.99 (2.40) C: 13.44 (2.10)	I: 67% C: 50%	NG	NG	NG
Fiallo-Scharer et al., 2019 [25]	I: 106 C: 108	Range: 8–16	I: 56% C: 56%	I: 82% C: 85%	I: 5.3 (3.1) C: 5.5 (3.5)	I: 57% C: 43%
Franklin et al., 2006 [26]	I: 31 C: 27	I: 13.07 (3.26) C: 12.67 (3.37)	I: 45% C: 37%	I: 96% C: 96%	I: 5.33 (3.73) C: 3.87 (3.91)	NG
Hanberger et al., 2013 [27]	I: 244 C: 230	I: 13.2 (3.7) C: 13.3 (3.7)	I: 52% C: 51%	NG	I: 4.9 (3.7) C: 5.1 (3.7)	I: 16% C: 16%
Henkemans et al., 2017 [28]	I: 16 C: 11	I: 10.00 (1.10) C: 12.55 (1.04)	I: 56% C: 45%	NG	I: 4.59 (2.31) C: 4.90 (2.41)	NG
Holmes et al., 2014 [11]	I: 136 C: 89	I: 12.95 (1.24) C: 12.73 (1.23)	I: 55% C: 46%	NG	I: 4.93 (2.95) C: 5.15 (3.16)	I: 45% C: 48%
Katz et al., 2014 [29]	I: 50 C: 51	I: 12.7 (2.2) C: 12.5 (2.3)	I: 58% C: 45%	I: 90% C: 98%	I: 6.5 (3.8) C: 5.7 (3.5)	I: 22% C: 20%
Kazeminezhad et al., 2018 [30]	I: 21 C: 24	Range 10–18	I: 58% C: 45%	NG	NG	NG
Mayer-Davis et al., 2018 [31]	I: 130 C: 128	I: 14.8 (1.1) C: 14.9 (1.1)	I: 45% C: 54%	NG	I: 6.48 (3.76) C: 6.39 (3.71)	I: 68% C: 73%
McGill et al., 2020 [32]	I: 77 C: 76	I: 14.9 (1.2) C: 15.1 (1.3)	I: 51% C: 53%	I: 78% C: 89%	I: 6.9 (3.9) C: 5.8 (3.5)	I: 61% C: 54%
Murphy et al., 2012 [33]	I: 158 C: 147	I: 13.1(1) C: 13.2 (2.0)	I: 53% C: 51%	I: 93% C: 91%	I: 5.5 (3.1) C: 5.6 (3.4)	I: 6% C: 7%
Nansel et al., 2015 [34]	I: 66 C: 70	I: 12.6 (2.7) C: 13.0 (2.5)	I: 47% C: 56%	I: 88% C: 93%	I: 5.6 (2.5) C: 6.3 (3.6)	I: 70% C: 69%
Nordfeldt et al., 2003 [35]	I: 111 C: 111	I: 12.7 (4.1) C: 12.5 (4.2)	I: 54% C: 54%	NG	I: 7.4 (3.9) C: 7.0 (4.1)	I: 34% C: 28%
Noyes et al., 2014 [36]	I: 190 C: 103	I: 12.4 (3.0) C: 12.7 (3.2)	I: 55% C: 52%	I: 94% C: 98%	I: 7.4 (3.8) C: 8.0 (3.9)	I: 12% C: 8%
Pais et al., 2021 [37]	I: 26 C: 24	I: 15.5 (2.4) C: 15.6 (2.4)	I: 46% C: 46%	NG	NG	I: 17% C: 19%
Price et al., 2016 [38]	I: 199 C: 197	I: 13.7 (1.4) C: 13.9 (1.6)	I: 54% C: 57%	I: 91% C: 93%	NG	NG
Rafeezadeh et al., 2019 [39]	I: 34 C: 34	I: 9.9 (1.4) C: 10.4 (1.2)	I: 44% C: 41%	NG	NG	NG
Skoufa et al., 2023 [40]	I: 42 C: 42	I: 12.6 (1.8) C: 12.6 (2.5)	NG	NG	I: 5.3 (3.1) C: 4.5 (3.0)	NG
Spiegel et al., 2012 [41]	I: 33 C: 33	I: 15.7 (3.4) C: 14.5 (1.8)	I: 24% C: 52%	I: 91% C: 91%	I: 5.5 (3.5) C: 5.6 (3.4)	I: 79% C: 82%
Svoren et al., 2003 [42]	I: 97 C: 108	I: 12.1 (2.4) C: 11.7 (2.6)	I: 58% C: 51%	NG	Overall: 5.3 (3.0)	I: 0% C: 0%
Whittemore et al., 2012 [13]	I: 167 C: 153	Overall: 12.3 (1.1)	I: 56% C: 55%	64.5%	NG	I: 59% C: 60%
Wang et al., 2010 [43]	I: 21 C: 23	I: 15.3 (1.4) C: 15.6 (1.7)	I: 57% C: 44%	I: 62% C: 74%	I: 6.7 (3.4) C: 7.6 (4.7)	NG
Wysocki et al., 2000 [44]	I: 38 C: 41	I: 14.5 (1.2) C: 14.3 (1.4)	I: 61% C: 51%	I: 78% C: 80%	I: 5.4 (3.8) C: 5.2 (3.8)	NG

Abbreviations: C, control; I, intervention; NG, not given/reported.

**Table 4:** Behavior change techniques used in included studies.

Studies	Goals and planning	Feedback and monitoring	Social support	Shaping knowledge	Natural consequences	Comparison of behavior	Associations	Repetition and substitution	Comparison of outcomes	Reward and threat	Regulation	Antecedents	Identity	Scheduled consequences	Self-belief	Covert learning
Alksir et al., 2022 [17]	x	x	—	x	—	—	—	—	—	—	—	—	—	—	x	—
Bakir et al., 2020 [18]	x	x	—	x	—	—	—	—	—	—	—	—	—	—	—	—
Bernier et al., 2018 [19]	—	—	—	x	—	—	—	—	—	—	—	—	—	—	—	—
Brorsson et al., 2019 [20]	x	—	—	x	—	—	—	—	—	—	—	—	x	—	—	—
Brown et al., 1997 [21]	—	—	x	x	—	—	—	—	—	—	—	—	—	—	—	—
Christie et al., 2016 [22]	x	x	—	x	x	—	—	—	—	—	—	—	—	—	—	—
Cook et al., 2002 [28]	x	x	x	x	—	—	—	—	—	—	—	—	—	—	x	—
Dłużniak-Gołaska et al., 2020 [24]	—	x	x	x	—	—	—	—	—	—	—	—	—	—	—	—
Fiallo-Scharer et al., 2019 [25]	x	x	x	x	—	—	—	—	—	—	—	—	—	—	—	—
Franklin et al., 2006 [26]	x	—	x	x	—	—	—	—	—	—	—	—	—	—	—	—
Whittemore et al., 2012 [13]	x	—	—	x	—	—	—	—	—	—	—	—	—	—	x	—
Hanberger et al., 2013 [27]	—	—	x	x	—	—	—	—	—	—	—	—	—	—	—	—
Henkemans et al., 2017 [28]	—	x	—	x	—	—	—	—	x	—	—	—	—	—	—	x
Holmes et al., 2014 [11]	x	x	x	x	—	—	—	—	—	—	—	—	—	—	—	—
Katz et al., 2014 [29]	—	x	x	x	—	—	—	—	x	—	—	—	—	—	—	—
Kazeminezhad et al., 2018 [30]	—	x	—	x	x	—	—	—	—	—	—	—	—	—	—	—
Mayer-Davis et al., 2018 [31]	x	—	x	x	—	—	—	—	—	—	—	—	—	—	—	—
McGill et al., 2020 [32]	x	—	—	x	—	—	—	—	—	—	—	—	x	—	x	—
Murphy et al., 2012 [33]	x	x	—	x	—	x	—	—	—	—	—	—	—	—	—	—
Nansel et al., 2015 [34]	x	x	—	x	x	—	—	—	x	—	—	—	—	—	x	—
Nordfeldt et al., 2003 [35]	—	—	—	x	—	x	—	—	—	—	—	—	—	—	—	—
Noyes et al., 2014 [36]	x	x	—	x	—	—	—	—	—	—	—	—	—	—	—	—
Pais et al., 2021[37]	—	—	—	x	—	—	—	—	—	—	—	—	—	—	—	—
Price et al., 2016 [38]	—	x	x	x	—	—	—	—	—	—	—	—	—	—	—	—
Rafeezadeh et al., 2019 [39]	—	—	—	x	—	—	x	x	—	—	—	—	—	—	—	x
Skoufa et al., 2023 [40]	—	—	x	x	—	—	—	x	—	—	—	—	—	—	—	—
Spiegel et al., 2012 [41]	—	x	—	x	—	—	—	—	—	—	—	—	—	—	—	—
Svoren et al., 2003 [42]	—	—	x	x	—	—	—	—	—	—	—	—	—	—	—	—
Wang et al., 2010 [43]	x	—	—	x	—	—	—	—	—	—	—	—	—	—	—	—
Wysocki et al., 2000 [44]	x	x	—	x	—	—	—	—	—	—	—	—	—	—	—	—

## Data Availability

The data that support the findings of this study are available from the corresponding author upon reasonable request.

## Supporting Information

Supporting Information S1 File 1: PRISMA reporting checklist.

Supporting Information S2 File 2: Search strategy developed using Ovid Medline.
