# Supplementary material for: Effectiveness of Educational and Psychoeducational Self-Management Interventions in Children and Adolescents With Type 1 Diabetes: A Systematic Review and Meta-Analysis
Source: Pediatr Diabetes. 2024 Oct 8;2024:2921845. doi: 10.1155/2024/2921845 (PMC12016993; doi:10.1155/2024/2921845)
Supplement: Supporting Information S2 — File 2: Search strategy developed using Ovid Medline. [file 2921845.f2.docx]

**Supplementary file 2: Search strategy**

Database: Ovid MEDLINE(R) ALL Search Strategy:

--------------------------------------------------------------------------------

1. Adolescent/
2. Child/
3. (young* adj2 (adult* or person* or individual* or people* or population* or man or mans or men or mens or woman* or women*or male or males or female*)).ti,ab.
4. minors.ti,ab.
5. (child or childs or children* or childhood* or childcare* or schoolchild*).ti,ab.
6. adolescen*.ti,ab.
7. juvenil*.ti,ab.
8. youth*.ti,ab.
9. teen*.ti,ab.
10. 1 or 2 or 3 or 4 or 5 or 6 or 7 or 8 or 9
11. Diabetes Mellitus, Type 1/
12. IDDM.ti,ab.
13. T1DM.ti,ab.
14. T1D.ti,ab.
15. (insulin dependent adj3 diabet*).ti,ab.
16. Typ* 1 diabet*.ti,ab.
17. 11 or 12 or 13 or 14 or 15 or 16
18. Patient Education as Topic/
19. Self-Management/
20. (psychoeducation* or psycho-education*).ti,ab.
21. self efficacy/
22. (patient* adj2 (education or advice or advis$ or instruct$ or educate or train$)).ti,ab.
23. ((Behavio?r* or lifestyle*) adj3 (change* or modif* or alter* or intervention* or technique*)).ti,ab.
24. self-management.ti,ab.
25. 18 or 19 or 20 or 21 or 22 or 23 or 24
26. 10 and 17 and 25
27. limit 26 to yr="1994 -Current"
